# Hospital admissions with influenza and impact of age and comorbidities on severe clinical outcomes in Brazil and Mexico

**DOI:** 10.1371/journal.pone.0273837

**Published:** 2022-11-10

**Authors:** Clotilde El Guerche-Séblain, Adrien Etcheto, Frédéric Parmentier, Mohammad Afshar, Alejandro E. Macias, Esteban Puentes, Viviane Gresset-Bourgeois, Meral Akcay, Audrey Petitjean, Laurent Coudeville

**Affiliations:** 1 Medical Influenza Franchise, Sanofi, Singapore, Singapore; 2 Epidemiology and Prevention Department, Hôpital Édouard Herriot, Université Claude Bernard Lyon 1, Lyon, France; 3 Ariana Pharmaceuticals, Paris, France; 4 Área De Microbiología, Departamento De Medicina, Universidad de Guanajuato, Guanajuato, Mexico; 5 Sanofi Latin America, Medical Franchise, Panama City, Panama; 6 Global Medical Strategy, Sanofi, Lyon, France; 7 Medical Influenza Franchise, Sanofi, Lyon, France; 8 Health Economics Value Assessment, Sanofi, Lyon, France; 9 Medical Evidence and Data Science, Sanofi, Lyon, France; Clinica Luganese Moncucco, SWITZERLAND

## Abstract

**Background:**

The risk of hospitalization or death after influenza infection is higher at the extremes of age and in individuals with comorbidities. We estimated the number of hospitalizations with influenza and characterized the cumulative risk of comorbidities and age on severe outcomes in Mexico and Brazil.

**Methods:**

We used national hospital discharge data from Brazil (SIH/SUS) from 2010–2018 and Mexico (SAEH) from 2010–2017 to estimate the number of influenza admissions using ICD-10 discharge codes, stratified by age (0–4, 5–17, 18–49, 50–64, and ≥65 years). Duration of hospital stay, admission to the intensive care unit (ICU), and in-hospital case fatality rates (CFRs) defined the severe outcomes. Rates were compared between patients with or without pre-specified comorbidities and by age.

**Results:**

A total of 327,572 admissions with influenza were recorded in Brazil and 20,613 in Mexico, with peaks period most years. In Brazil, the median hospital stay duration was 3.0 days (interquartile range, 2.0–5.0), ICU admission rate was 3.3% (95% CI, 3.2–3.3%), and in-hospital CFR was 4.6% (95% CI, 4.5–4.7). In Mexico, the median duration of stay was 5.0 days (interquartile range, 3.0–7.0), ICU admission rate was 1.8% (95% CI, 1.6–2.0%), and in-hospital CFR was 6.9% (95% CI, 6.5–7.2). In Brazil, ICU admission and in-hospital CFR were higher in adults aged ≥50 years and increased in the presence of comorbidities, especially cardiovascular disease. In Mexico, comorbidities increased the risk of ICU admission by 1.9 (95% CI, 1.0–3.5) and in-hospital CFR by 13.9 (95% CI, 8.4–22.9) in children 0–4 years.

**Conclusion:**

The SIH/SUS and SAEH databases can be used to estimate hospital admissions with influenza, and the disease severity. Age and comorbidities, especially cardiovascular disease, are cumulatively associated with more severe outcomes, with differences between countries. This association should be further analyzed in prospective surveillance studies designed to support influenza vaccination strategy decisions.

## Introduction

Each year, seasonal influenza is associated with up to 3–5 million cases of severe illness and approximately 290–650 thousand deaths worldwide [1]. Although all persons are at risk of infection, the risk of severe influenza that leads to hospitalization or death is higher for children <5 years of age, older adults, pregnant women, and individuals with underlying conditions such as immunodeficiencies, asthma, and chronic heart or lung diseases [1–3]. This is especially true for low- and middle-income countries [3]. However, evaluating the true burden of severe influenza in these countries is challenging due to non-systematic laboratory testing of patients hospitalized with acute respiratory infections, limited access to influenza diagnostic tests, and/or lack of data on specific risk factors. The World Health Organization (WHO) recommends seasonal flu vaccination for those who are most at-risk for morbidity and mortality, particularly the elderly and those with underlying health conditions regardless of age, such as diabetes, hypertension, human immunodeficiency virus infection, asthma and other chronic heart or lung diseases [1]. Although the vast majority of countries recommend vaccination of the at-risk population, vaccination coverage rates (VCR) for this group remains low in many countries (below the target of 75%), including more developed countries [4].

Brazil is the largest country in Latin America. The annual number of influenza cases was estimated to be between 4.2 and 6.4 million cases in 2008, and influenza-like illness led to 4.4% to 16.9% of hospital admissions between 2000 and 2008 [5]. Excess mortality associated with influenza was documented in Southern Brazil with 1.4/100,000 person-years for all ages, and 9.2/100,000 person-years for adults ≥60 years of age between 1980 and 2008 [6].

In Mexico the annual number of confirmed influenza cases was estimated to be between 0.8 and 1.1 million in 2008 [5]. All-cause influenza-associated mortality in Mexico was estimated as 20.3 deaths/100,000 for the 2010–2015 period [7].

Although the factors of age, comorbidities, and virus subtypes have been independently associated with mortality and intensive care unit (ICU) admission in hospitalized patients [8, 9], studies evaluating the cumulative risk of age and comorbidities in patients hospitalized with influenza are scarce in both countries. There are large publicly-available administrative hospital databases in both Brazil and Mexico that have been previously used to estimate burden of dengue at hospitals in Mexico or in-hospital mortality in Brazil, but not yet explored for seasonal influenza disease and for severe outcomes other than in-hospital mortality [10–12]. Using these databases, our study objectives were to estimate the number of hospital admissions with influenza in Brazil and Mexico, compare the associated severe outcomes in patients with or without comorbidities, and estimate the cumulative effect of comorbidities and age on these outcomes.

## Methods

### Study design

This cross-sectional study was based on national administrative hospital discharge data from Brazil between 2010 and 2018, and Mexico between 2010 and 2017.

### Data sources

Anonymized data from two publicly available administrative hospital discharge databases were used in this study. The Brazilian Hospital Information System of the Unified Health System (SIH/SUS) included discharge data from 5,930 public and private hospitals, which covered 88.4% of all hospitals in Brazil in 2014 [13]. This represented approximately 75% of the hospitalizations as only hospitalizations financed by the public healthcare system (SUS) are registered in this database. Approximately 103 million hospital admissions were recorded in the SIH/SUS database between January 1, 2010 and December 31, 2018. The Mexican Automated Subsystem for Hospital Discharges (SAEH) is the main hospital discharge database for all Ministry of Health hospitals in Mexico. It includes 859 hospitals, and represents approximately 40% of the hospitalizations in Mexico [14]. Approximately 23 million admissions were recorded in the SAEH database between January 1, 2010 and December 31, 2017. Approval by ethics committees and patient consent were not required because only anonymized data were extracted from pre-existing national hospital databases for analysis.

### Definitions of influenza cases, comorbidities, and severe outcomes

From these databases, we selected all hospital admissions with a primary or secondary discharge diagnosis of influenza. Discharge diagnoses were based on the International Classification of Diseases, 10^th^ Revision (ICD-10) codes, version 2016 [15]. Influenza cases were defined using the following codes: J09 (influenza due to identified zoonotic or pandemic influenza virus), J10 (influenza due to identified seasonal influenza virus), J11 (influenza, virus not identified), and J12.9 (viral pneumonia, unspecified). In Brazil, the number of fields available for reporting secondary discharge diagnostic codes in the SIH/SUS database varied over time. Each entry could contain only one secondary code from 2010 to 2014, and up to nine codes from 2015 onwards. By contrast, in Mexico each entry in the SAEH database contained unlimited fields for secondary diagnoses over the whole study period. For consistency, we limited this analysis to the first nine secondary codes in Mexico.

Comorbidities considered to increase the risk of influenza complications were selected using the published literature and WHO recommendations [3, 16], and grouped into four predefined categories: cardiovascular disease (ICD-10 codes: I10–I13, I15, I20–I25, I26–I28, I30–I52, I60–I74, I77–I89, I95, I97–I99), chronic obstructive pulmonary disease (COPD; J40–J47), diabetes (E10, E11, E13, E14), and immunodeficiencies (D71, D80–D84, D89).

Three outcome measures were defined to assess severe influenza cases: hospital stay duration, admission to ICU, and in-hospital mortality.

### Analyses

Time series for weekly hospital admissions with influenza were calculated using the Serfling regression method [17] to model a basic level of influenza impact on each week outside of the epidemic period in each country. Epidemic peaks were defined as periods for which the observed admissions exceeded the upper 90% confidence limit of those predicted by the model (i.e., the epidemic threshold) for at least two consecutive weeks. Discharge records were stratified by patient age: 0–4 years, 5–17 years, 18–49 years, 50–64 years, and ≥65 years.

ICU admission rates were calculated as the proportion of cases that were admitted to ICU during a given hospitalization. In-hospital mortality was assessed by case-fatality rates (CFRs), calculated as the proportion of influenza cases that died during hospitalization. Only in-hospital deaths during a given hospitalization episode were considered. The duration of hospital stay was calculated as the difference between the admission date and the discharge date.

Relative risks (RRs), with associated 95% confidence intervals (CIs), were calculated as the ratios of the ICU admission rates or CFRs in cases with pre-defined comorbidities (cardiovascular, COPD, diabetes, immunodeficiency) to those without comorbidities. Finally, rates of severe outcomes for patients with predefined comorbidity with or without influenza were calculated. For each predefined comorbidity, association between having influenza, and a severe outcome was tested using a Fisher’s exact test. P values ≤0.05 were considered statistically significant.

## Results

### Hospital admissions with influenza

In Brazil, 327,572 admissions with discharge ICD-10 codes associated with influenza were recorded in the SIH/SUS database between 2010 and 2018 (0.3% of the total admissions). In Mexico, 20,613 of such admissions were recorded in the SAEH database between 2010 and 2017 (0.1% of the total admissions) (**Table 1**). In Brazil, the most frequent ICD-10 code used for influenza admissions was J11 (influenza, virus not identified; 49.5%), followed by J12.9 (31.1%), J10 (19.4%), and J09 (0.04%), whereas in Mexico, the most frequent influenza code was J12.9 (viral pneumonia, unspecified; 63.8%), followed by J11 (25.4%), J09 (5.8%), and J10 (5.0%). In both countries hospital admissions associated with influenza occurred mostly in children aged <5 years (29.8% of admissions in Brazil and 58.4% in Mexico). Such admissions were also frequent in Brazilian adults aged ≥65 years (25.9%), but not in Mexican adults aged ≥65 years (7.7%). One or more comorbidity was present in 1% (95% CI, 0.9–1.1) of admissions with influenza in Brazil and 14.3% (95% CI, 13.8–14.7) of those in Mexico. These comorbidities were more frequent among admissions in patients aged ≥50 years.

**Table 1:** Patient characteristics and severe outcome estimates.

	Brazil (N = 103,189,553)						Mexico (N = 22,723,448)					
	0−4 y (N = 10,261,531)	5−17 y (N = 10,016,201)	18−49 y (N = 47,400,703)	50−64 y (N = 16,350,052)	≥65 y (N = 19,160,991)	Total (N = 103,189,553)	0−4 y (N = 2,234,222)	5−17 y (N = 2,818,694)	18−49 y (N = 13,640,674)	50−64 y (N = 2,106,794)	≥65 y (N = 1,908,799)	Total (N = 22,723,448)
Patients admitted with influenza, N (% of total admissions) [95% CI]	97,487 (0.9%) [0.9–1.0]	39,265 (0.4%) [0.4–0.4]	67,587 (0.1%) [0.1–0.1]	38,285 (0.2%) [0.2–0.2]	84,948 (0.4%) [0.4–0.4]	327,572 (0.3%) [0.3–0.3]	12,048 (0.5%) [0.5–0.5]	1,682 (0.1%) [0.1–0.1]	3,683 (0.0%) [0.0–0.0]	1,613 (0.1%) [0.1–0.1]	1,587 (0.1%) [0.1–0.1]	20,613 (0.1%) [0.1–0.1]
Admissions with influenza by age group, %	29.8%	12.0%	20.6%	11.7%	25.9%	100%	58.4%	8.2%	17.9%	7.8%	7.7%	100%
Predefined comorbidities, n (% of influenza admissions) [95% CI]	796 (0.8%) [0.8–0.9]	163 (0.4%) [0.4–0.5]	403 (0.6%) [0.6–0.7]	564 (1.5%) [1.3–1.6]	1,408 (1.7%) [1.6–1.7]	3,364 (1.0%) [0.9–1.1]	578 (4.8%) [4.4–5.2]	209 (12.4%) [10.8–14.0]	675 (18.3%) [17.1–19.6]	673 (41.7%) [39.3–44.1]	804 (50.7%) [48.2–53.1]	2,939 (14.3%) [13.8–14.7]
Diabetes, n (% of comorbidities) [95% CI]	6 (0.7%) [0.1–1.3]	9 (5.5%) [2.0–9.0]	42 (10.4%) [7.4–13.4]	92 (16.3%) [13.3–19.4]	156 (11.1%) [9.4–12.7]	305 (9.1%) [8.1–10.0]	5 (0.9%) [0.1–1.6]	3 (1.4%) [0.0–3.0]	296 (43.8%) [40.1–47.6]	334 (49.6%) [45.8–53.4]	259 (32.2%) [28.9–35.4]	897 (30.5%) [28.9–32.2]
Cardiovascular disease, n (% of comorbidities) [95% CI]	37 (4.6%) [3.2–6.1]	9 (5.5%) [2.0–9.0]	165 (40.9%) [36.1–45.7]	300 (53.2%) [49.1–57.3]	788 (56.0%) [53.4–58.6]	1,299 (38.6%) [37.0–40.3]	187 (32.3%) [28.5–36.2]	36 (17.2%) [12.1–22.3]	287 (42.5%) [38.8–46.2]	381 (56.6%) [52.9–60.4]	463 (57.6%) [54.2–61.0]	1,354 (46.1%) [44.3–47.9]
COPD, n (% of comorbidities) [95% CI]	753 (94.6%) [93.0–96.2]	143 (87.7%) [82.7–92.7]	212 (52.6%) [47.7–57.5]	250 (44.3%) [40.2–48.4]	578 (41.1%) [38.5–43.6]	1,936 (57.6%) [55.9–59.2]	366 (63.2%) [59.4–67.3]	168 (80.4%) [75.0–85.8]	206 (30.5%) [27.0–34.0]	173 (25.7%) [22.4–29.0]	367 (45.6%) [42.2–49.1]	1,280 (43.6%) [41.8–45.3]
Immunodeficiency, n (% of comorbidities) [95% CI]	3 (0.3%) [0.0–0.8%]	3 (1.8%) [0.0–3.9]	0 (0.0%) [0.0–0.9]	0 (0.0%) [0.0–0.7]	0 (0.0%) [0.0–0.3]	6 (0.2%) [0.1–0.4]	26 (4.5%) [2.8–6.2]	3 (1.4%) [0.0–3.0]	1 (0.1%) [0.0–0.4]	0 (0.0%) [0.0–0.6]	0 (0.0%) [0.0–0.5]	30 (1.0%) [0.7–1.5]
Median duration of stay, days [IQR]	3.0 [2.0–5.0]	3.0 [2.0–4.0]	3.0 [2.0–4.0]	3.0 [2.0–5.0]	4.0 [3.0–6.0]	3.0 [2.0–5.0]	4.0 [3.0–7.0]	4.0 [2.0–6.0]	5.0 [2.0–9.0]	6.0 [3.0–11.0]	5.0 [3.0–9.0]	5.0 [3.0–7.0]
ICU admission, n (% of influenza admissions) [95% CI]	2,484 (2.5%) [2.4–2.6]	458 (1.2%) [1.1–1.3]	2,044 (3.0%) [2.9–3.1]	1,835 (4.8%) [4.6–5.0]	3,844 (4.5%) [4.4–4.7]	10,665 (3.3%) [3.2–3.3]	151 (1.2%) [1.1–1.4]	23 (1.4%) [0.8–1.9]	139 (3.8%) [3.2–4.4]	49 (3.0%) [2.2–3.9]	18 (1.1%) [0.6–1.7]	370 (1.8%) [1.6–2.0]
In-hospital death, n (% of influenza admissions) [95% CI]	392 (0.4%) [0.4–0.4]	150 (0.4%) [0.3–0.4]	1,648 (2.4%) [2.3–2.6]	2,305 (6.0%) [5.8–6.3]	10,694 (12.6%) [12.4–12.8]	15,189 (4.6%) [4.5–4.7]	162 (1.3%) [1.1–1.6]	45 (2.7%) [1.9–3.4]	478 (13.0%) [11.9–14.1]	362 (22.4%) [20.4–24.5]	370 (23.3%) [21.2–25.4]	1,417 (6.9%) [6.5–7.2]

CI, confidence interval; COPD, chronic obstructive pulmonary disease; ICU, intensive care unit; IQR, interquartile range

**Fig 1:**
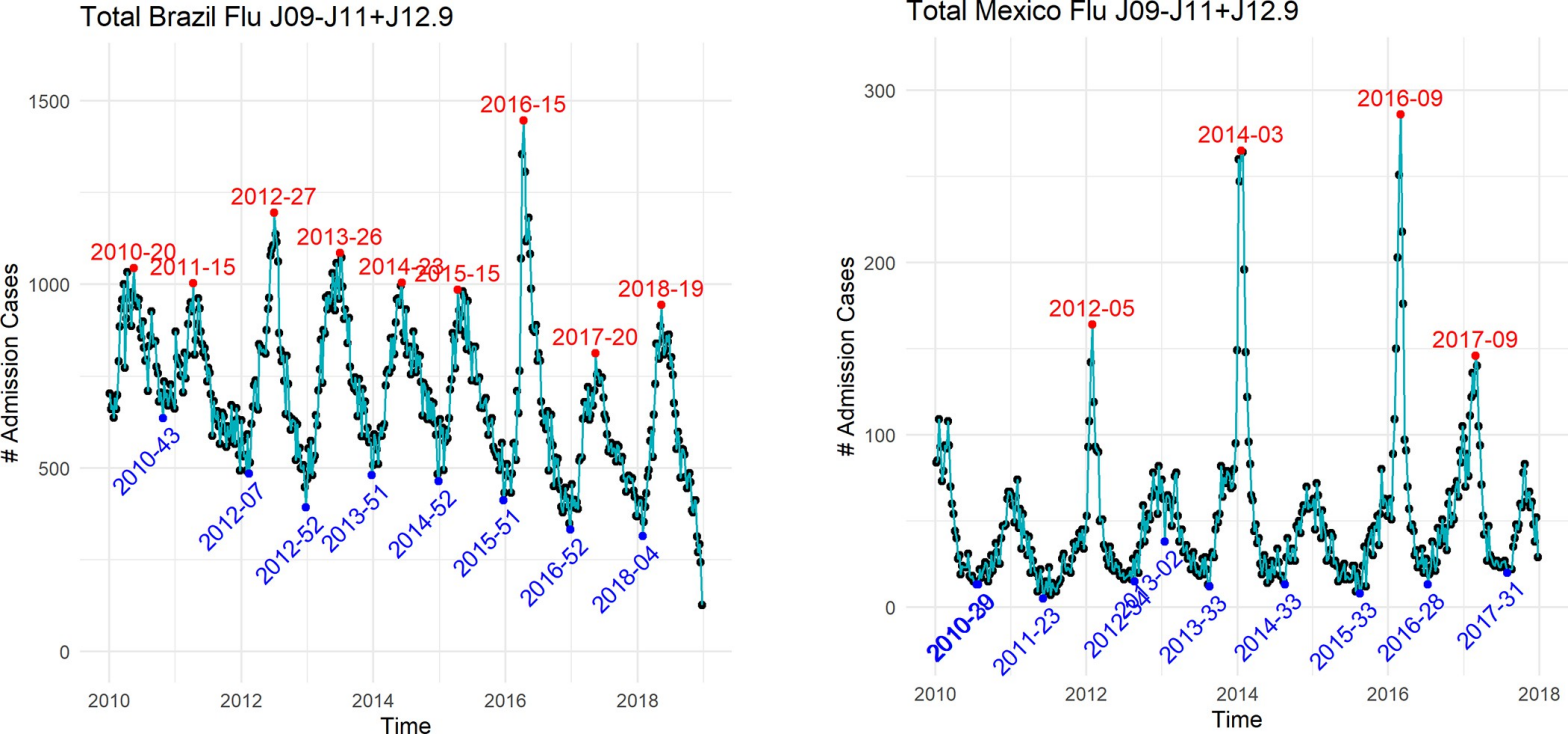
Time-series of hospital admissions in Brazil and Mexico. Time-series of hospital admissions (A) in Brazil between 2010 and 2018 and (B) in Mexico between 2010 and 2017. The weeks of admission peaks are indicated in red and the weeks of admission nadirs are indicated in blue.

In both countries, weekly hospital admissions with influenza increased during specific periods of the year with an observable annual epidemic peak (**Fig 1**). In Brazil, weekly admissions ranged between 250 and 1,500, with an epidemic period varying from Week 15 (April) to Week 29 (July), except in 2017 in which the admissions remained below the epidemic threshold (**Fig 1A**). In Mexico, the weekly admissions ranged between 20 and 290 with an epidemic period varying from Week 03 (January) in 2014 to Week 09 (End February) in 2016 (**Fig 1B**).

### Prevalence of severe outcomes

Patients admitted with influenza in Brazil were hospitalized for a median duration of 3.0 days (interquartile range [IQR], 2.0–5.0), with 3.3% (95% CI, 3.2–3.3%) of patients being admitted to the ICU (**Table 1**). In Mexico, patients were hospitalized for a median duration of 5.0 days (IQR, 3.0–7.0), with 1.8% (95% CI, 1.6–2.0%) of patients being admitted to ICU (**Table 1**). In-hospital death was reported for 4.6% (95% CI, 4.5–4.7) of all admissions with influenza in Brazil and 6.9% (95% CI, 6.5–7.2) of those in Mexico. Most deaths occurred among patients ≥50 years of age, with 12,999/15,189 (85.6%) in-hospital deaths occurring in these patients in Brazil and 732/1,417 (51.7%) in Mexico.

### Risk of severe outcomes by age and comorbidity

**Fig 2:**
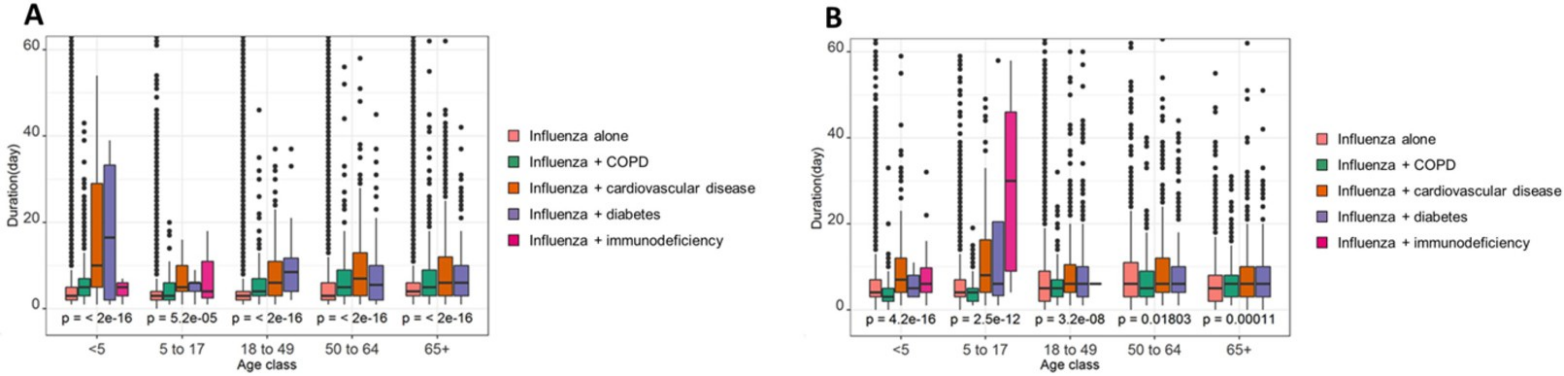
Duration of hospital stay among hospital admissions with influenza by age and comorbidity. (**A**) In Brazil. (**B**) In Mexico. The box plots represent the medians and the interquartile ranges.

The duration of hospital stay was generally longer in the presence of comorbidities, particularly for children <5 years of age in Brazil, and those 5–18 years of age in Mexico (**Fig 2**). In Brazil, the longest duration of hospital stay with influenza was for children <5 years of age with diabetes (median 16.5 days [IQR, 2.0–33.3]), and in Mexico it was for children 5–17 years of age with cardiovascular disease (median 8.0 days [IQR, 4.0–15.3]) (**S1 Table**).

**Fig 3:**
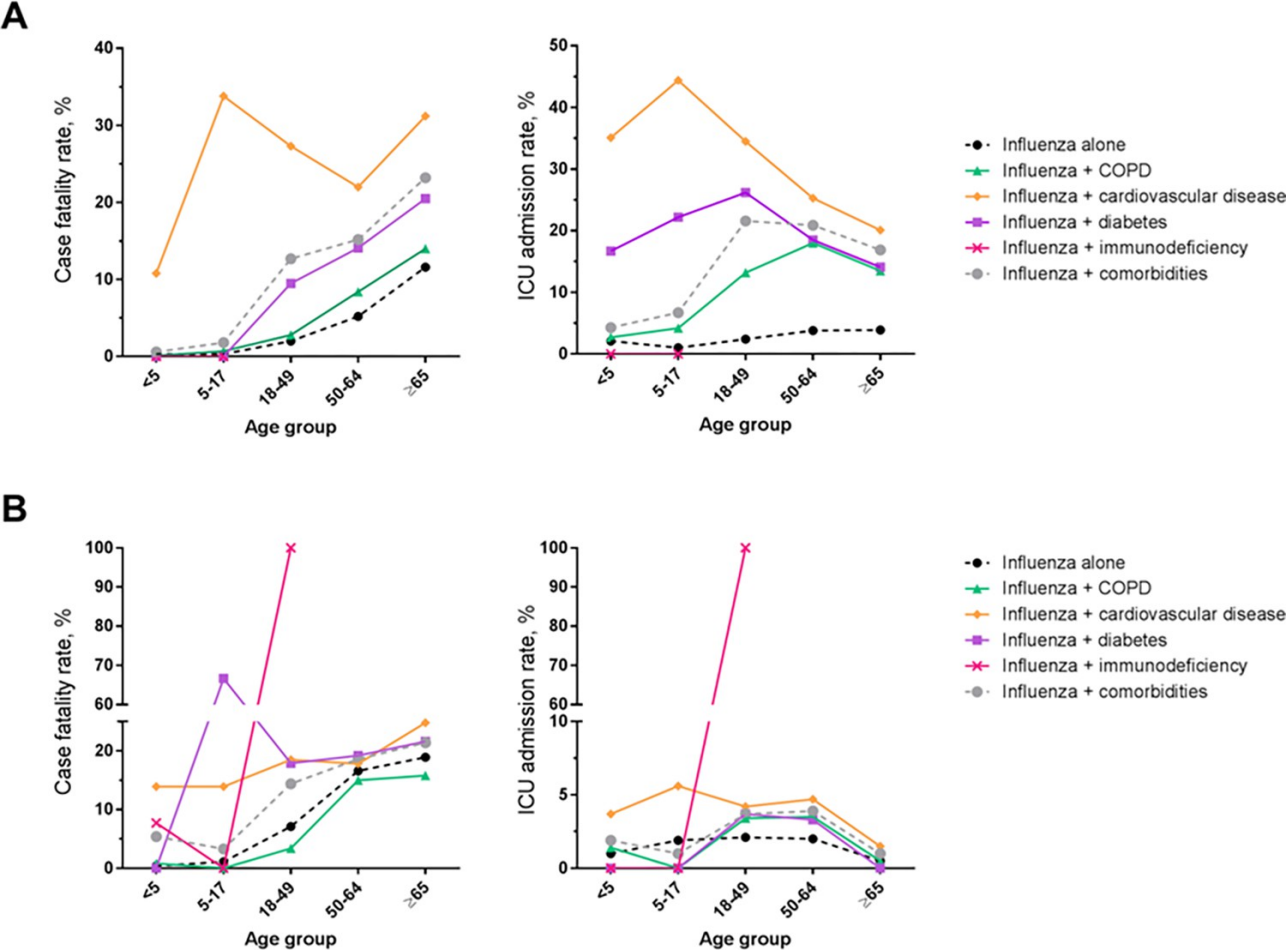
Influenza severity among Brazilian and Mexican hospital admissions with influenza by age and comorbidity. Case fatality rates (left) and ICU admissions (right) for hospitalized influenza patients, with and without comorbidities, stratified by age group, in (**A**) Brazil and (**B**) Mexico. COPD, chronic obstructive pulmonary disease. Only subgroups including more than 10 admissions (see Table 1) are shown.

In Brazil, ICU admission rates and in-hospital CFRs increased with both age and with the presence of comorbidities (**Fig 3A**). ICU admissions in children aged 0–4 years were 2.1% (95% CI, 2.0–2.2) of those without comorbidities and 4.3% (95% CI, 2.9–5.7) of those with comorbidities, and in adults ≥65 years ICU admissions were 3.9% (95% CI, 3.8–4.0) of those without comorbidities and 16.9% (95% CI, 14.9–18.9) of those with comorbidities. Across age groups, the highest proportions of ICU admissions were in patients with cardiovascular disease, ranging from 20.1% (95% CI, 17.3–22.8) of patients ≥65 years to 44.4% (95% CI, 12.0–76.9) of patients aged 18–49 years. The presence of comorbidities increased in-hospital CFRs from 0.3% (95% CI, 0.3–0.3) to 0.6% (95% CI, 0.3–1.4) of children aged 0–4 years and from 11.6% (95% CI, 11.4–11.8) to 23.2% (95% CI, 20.9–25.4) of adults ≥65 years, compared to patients without comorbidities. This suggests a cumulative role of age and comorbidities on mortality in patients hospitalized with influenza in Brazil. As with ICU admissions, the highest CFRs were for patients with cardiovascular diseases in all age groups. In line with these results, the RRs of ICU admissions and in-hospital CFR in patients with comorbidities to those without comorbidities was highest for cardiovascular disease in all age groups, and especially in children 5–17 years of age (RR, 101.4 [95% CI, 39.6–259.8]) (**Table 2**).

**Table 2:** Relative risk of ICU admission and in-hospital case fatality rate for patients with predefined comorbidities admitted with influenza.

	ICU admission rate, RR (95% CI)					In-hospital case fatality rate, RR (95% CI)				
	0−4 years	5−17 years	18−49 years	50−64 years	≥65 years	0−4 years	5−17 years	18−49 years	50−64 years	≥65 years
**Brazil**										
Any predefined comorbidity	2.0 (1.4–2.8)	6.7 (3.8–12.0)	9.0 (7.4–10.9)	5.4 (4.62–6.42)	4.4 (3.9–4.9)	2.2 (0.9–5.3)	5.6 (1.8–17.4)	6.2 (4.8–8.1)	2.9 (2.4–3.6)	2.0 (1.8–2.2)
Cardiovascular disease	16.3 (10.3–25.4)	44.3 (21.2–92.5)	14.4 (11.6–17.9)	6.6 (5.4–8.1)	5.2 (4.5–6.0)	37.8 (14.9–96.1)	101.4 (39.6–259.8)	13.4 (10.4–17.3)	4.2 (3.4–5.3)	2.7 (2.4–3.0)
COPD	1.2 (0.8–1.9)	4.2 (1.9–9.2)	5.5 (3.9–7.8)	4.7 (3.6–6.1)	3.5 (2.8–4.3)	0.5 (0.1–3.3)	2.1 (0.3–15.1)	1.4 (0.6–3.1)	1.6 (1.1–2.4)	1.2 (0.9–1.5)
Diabetes	7.7 (1.3–46.4)	22.1 (6.59–75.4)	11.0 (6.6–18.2)	4.8 (3.13–7.4)	3.6 (2.5–5.4)	N/A	N/A	4.7 (1.8–11.9)	2.7 (1.6–4.5)	1.8 (1.3–2.4)
Immunodeficiency	N/A	N/A	N/A	N/A	N/A	N/A	N/A	N/A	N/A	N/A
**Mexico**										
Any predefined comorbidity	1.9 (1.0–3.5)	0.5 (0.1–2.2)	1.7 (1.0–3.0)	2.0 (0.9–4.1)	2.0 (0.42–9.2)	13.9 (8.4–22.9)	2.9 (1.1–7.8)	2.0 (1.6–2.7)	1.1 (0.9–1.4)	1.1 (0.9–1.4)
Cardiovascular disease	3.7 (1.7–7.9)	2.9 (0.7–12.3)	2.0 (1.0–3.8)	2.4 (1.1–5.3)	3.0 (0.6–14.4)	35.9 (21.5–60.1)	12.1 (4.3–34.4)	2.6 (1.9–3.6)	1.1 (0.8–1.4)	1.3 (1.0–1.7)
COPD	1.3 (0.55–3.33)	N/A	1.6 (0.71–3.61)	1.8 (0.6–4.9)	1.1 (0.1–7.6)	2.1 (0.6–6.9)	N/A	0.5 (0.2–1.0)	0.9 (0.6–1.4)	0.8 (0.6–1.1)
Diabetes	N/A	N/A	1.7 (0.9–3.5)	1.7 (0.7–4.0)	N/A	N/A	58.3 (20.8–163.4)	2.5 (1.9–3.5)	1.1 (0.9–1.6)	1.1 (0.8–1.6)
Immunodeficiency	N/A	N/A	47.1 (32.6–67.9)	N/A	N/A	19.9 (5.0–79.2)	N/A	14.2 (11.6–17.2)	N/A	N/A

COPD, chronic obstructive pulmonary disease; ICU, intensive care unit; N/A, not applicable. RR, relative risk.

In Mexico, ICU admissions rates ranged between 0.5% (95% CI, 0.1–1.8) and 2.1% (95% CI, 1.5–3.1) of hospitalized patients without comorbidities and between 1.0% (95% CI, 0.0–2.3) and 3.9% (95% CI, 2.4–5.3) of those with comorbidities (**Fig 3B**). The only exception was for the three children 5–17 years of age with immunodeficiency who were all admitted to ICU. In patients ≥50 years of age, the presence of any comorbidity did not significantly increase ICU admission rates, except cardiovascular disease in patients 50–64 years of age (RR, 2.40 [95% CI, 1.09–5.28]) (**Table 2**). The presence of any of the pre-specified comorbidities did not significantly increase the RR of in-hospital CFR in adults ≥50 years of age in Mexico. However, compared to patients without comorbidities, in-hospital CFR increased in the presence of comorbidities from 0.4% (95% CI, 0.3–0.6) to 5.4% (95% CI, 3.5–7.2) of children 0–4 years, but varied only from 18.9% (95% CI, 15.3–23.0) to 21.4% (95% CI, 18.6–24.2) of those ≥65 years (**Fig 3**). The RRs of in-hospital CFR in patients with comorbidities to those without comorbidities were highest in children, with a maximum for children 5–17 years of age with diabetes (58.3 [95% CI, 20.8–163.4]) (**Table 2**).

To get further insight into the role of comorbidities in severe influenza, we compared the rates of severe outcomes for each predefined comorbidity among hospital admissions with or without influenza. Both in Brazil (**Table 3**) and in Mexico (**Table 4**), all predefined comorbidities were significantly associated with higher in-hospital CFR and ICU admission rates in at least one age group in patients hospitalized with influenza, compared to patients with any disease other than influenza.

**Table 3:** Admissions to intensive care unit and in-hospital case fatality rates by age and predefined comorbidity in Brazil.

	ICU admission rate (n; %)					In-hospital case fatality rate (n; %)				
Comorbidity	0−4 years	5−17 years	18−49 years	50−64 years	≥65 years	0−4 years	5−17 years	18−49 years	50−64 years	≥65 years
Any predefined comorbidity, n (%)										
n with influenza	796	163	403	564	1,408	796	163	403	564	1,408
n with other diagnoses	67,629	39,307	294,694	538,668	866,422	67,629	39,307	294,694	538,668	866,422
% severe outcomes with influenza (95% CI)	4.3% (2.9–5.7)	6.7% (2.9–10.6)	21.6% (17.6–25.6)	20.9% (17.6–24.1)	16.9% (14.9–18.9)	0.6% (0.3–1.4)	1.8% (0.0–3.9)	12.7% (9.5–15.9)	15.2% (12.3–18.0)	23.2% (20.9–25.4)
% severe outcomes with other diagnoses (95% CI)	11.9% (11.6–12.1)	12.0% (11.7–12.4)	16.6% (16.5–16.8)	19.0% (18.9–19.1)	17.9% (17.8–17.9)	4.3% (4.1–4.4)	3.3% (3.1–3.5)	9.6% (9.5–9.7)	13.3% (13.2–13.4)	23.2% (23.1–23.3)
p-value	<0.001	0.039	0.009	0.250	0.365	<0.001	0.383	0.042	0.183	0.975
Diabetes										
n with influenza	6	9	42	92	156	6	9	42	92	156
n with other diagnoses	803	4,469	51,047	114,531	166,152	803	4,469	51,047	114,531	166,152
% severe outcomes with influenza (95% CI)	16.7% (0.0–46.5)	22.2% (0.0–49.4)	26.2% (12.9–39.5)	18.5% (10.5–26.4)	14.1% (8.6–18.6)	0.0% (0–39.2)	0.0% (0–29.9)	9.5% (0.6–18.4)	14.1% (7.0–21.2)	20.5% (14.2–26.8)
% severe outcomes with other diagnoses (95% CI)	17.9% (15.3–20.6)	21.3% (20.1–22.5)	12.3% (12.0–12.5)	12.6% (12.4–12.8)	12.8% (12.7–13.0)	4.7% (3.2–6.2)	1.9% (1.5–2.3)	6.1% (5.9–6.3)	9.1% (9.0–9.3)	16.9% (16.7–17.1)
p-value	0.999	0.999	0.015	0.114	0.632	0.999	0.999	0.323	0.102	0.240
Cardiovascular disease										
n with influenza	37	9	165	300	788	37	9	165	300	788
n with other diagnoses	11,809	12,606	244,360	462,064	750,335	11,809	12,606	244,360	462,064	750,335
% severe outcomes with influenza (95% CI)	35.1% (19.8–50.5)	44.4% (12.0–76.9)	34.5% (27.3–41.8)	25.3% (20.4–30.3)	20.1% (17.3–22.8)	10.8% (0.8–20.8)	33.3% (2.5–64.1)	27.4% (20.5–34.1)	22.0% (17.3–26.7)	31.2% (28.0–34.5)
% severe outcomes with other diagnoses (95% CI)	46.7% (45.8–47.6)	22.7% (22.0–22.5)	17.9% (17.7–18.0)	20.4% (20.3–20.5)	18.8% (18.8–18.9)	22.9% (22.1–23.7)	9.3% (8.8–9.8)	10.4% (10.2–10.5)	13.5% (13.4–13.6)	23.2% (23.1–23.3)
p-value	0.188	0.126	<0.001	0.038	0.387	0.114	0.044	<0.001	<0.001	<0.001
COPD										
n with influenza	753	143	212	250	578	753	143	212	250	578
n with other diagnoses	54,879	22,151	19,169	40,947	90,295	54,879	22,151	19,169	40,947	90,295
% severe outcomes with influenza (95% CI)	2.7% (1.5–3.8)	4.2% (0.9–7.5)	13.2% (8.6–17.8)	18.0% (13.2–22.8)	13.5% (10.7–16.3)	0.1% (0.0–0.4)	0.1% (0.0–2.1)	2.8% (0.6–5.1)	8.4% (5.0–11.8)	14.0% (11.2–16.8)
% severe outcomes with other diagnoses (95% CI)	4.4% (4.2–4.6)	4.4% (4.1–4.6)	10.2% (9.8–10.7)	15.5% (15.2–15.9)	14.8% (14.6–15.1)	0.3% (0.3–0.4)	0.3% (0.3–0.4)	5.8% (5.5–6.2)	15.6% (15.3–16.0)	26.1% (25.8–26.4)
p-value	0.016	0.999	0.17	0.293	0.411	0.526	0.379	0.074	0.001	<0.001
Immune deficiency										
n with influenza	3	3	0	0	0	3	3	0	0	0
n with other diagnoses	489	500	643	210	201	489	500	643	210	201
% severe outcomes with influenza	0.0%	0.0%	N/A	N/A	N/A	0.0%	0.0%	N/A	N/A	N/A
% severe outcomes with other diagnoses (95% CI)	10.3% (11.5–17.8)	8.9% (6.4–11.4)	8.9% (6.7–11.1)	10.5% (6.3–14.6)	8.0% (4.2–11.7)	4.7% (3.2–6.2)	2.2% (0.1–3.5)	7.8% (5.7–9.8)	18.1% (12.9–23.3)	16.4% (11.3–21.5)
p-value	0.999	0.999	N/A	N/A	N/A	0.999	0.999	N/A	N/A	N/A

CI, confidence interval; COPD, chronic obstructive pulmonary disease; ICU, intensive care unit; N/A, not applicable. For each comorbidity, the denominator (n) of the percentages is the number of patients with influenza + the considered comorbidity or the number of patients without influenza + the considered comorbidity.

**Table 4:** Admissions to intensive care unit and in-hospital case fatality rates by age and predefined comorbidity in Mexico.

	ICU admission rate (n; %)					In-hospital case fatality rate (n; %)				
Comorbidity	0−4 years	5−17 years	18−49 years	50−64 years	≥65 years	0−4 years	5−17 years	18−49 years	50−64 years	≥65 years
Any predefined comorbidity										
n with influenza	578	209	675	673	804	578	209	675	673	804
n other diagnoses	44,742	36,028	355,876	415,516	518,732	44,742	36,028	355,876	415,516	518,732
% severe outcomes with influenza (95% CI)	1.9% (0.7–3.0)	1.0% (0.0–2.3)	3.7% (2.3–5.1)	3.9% (2.4–5.3)	1.0% (0.3–1.7)	5.4% (3.5–7.2)	3.3% (0.9–5.8)	14.4% (11.7–17.0)	18.6% (15.6–21.5)	21.4% (18.6–24.2)
% severe outcomes with other diagnoses (95% CI)	2.1% (2.0–2.2)	1.3% (1.2–1.4)	1.1% (1.0–1.1)	0.8% (0.8–0.9)	0.6% (0.6–0.7)	12.6% (12.3–12.9)	6.5% (6.2–6.7)	7.3% (7.2–7.3)	10.3% (10.2–10.4)	16.5% (16.4–16.6)
p-value	0.884	0.999	<0.001	<0.001	0.188	<0.001	0.066	<0.001	<0.001	<0.001
Diabetes										
n with influenza	5	3	296	334	259	5	3	296	334	259
n other diagnoses	696	7026	182,889	271,783	246,075	696	7,026	182,889	271,783	246,075
% severe outcomes with influenza (95% CI)	0.0% (0.0–43.4)	0.0% (0.0–56.1)	3.7% (1.6–5.9)	3.3% (1.4–5.2)	0.0% (0.0–1.6)	0.0% (0.0–43.4)	66.7% (13.3–100.0)	17.9% (13.5–22.3)	19.2% (14.9–23.4)	21.6% (16.6–26.6)
% severe outcomes with other diagnoses (95% CI)	1.0% (0.3–1.8)	1.8% (1.5–2.1)	1.0% (0.9–1.0)	0.6% (0.6–0.7)	0.6% (0.5–0.6)	6.3% (4.5–8.1)	3.1% (2.7–3.5)	6.7% (6.6–6.8)	9.4% (9.3–9.6)	14.1% (13.9–14.2)
p-value	0.999	0.999	<0.001	<0.001	0.412	0.999	0.003	<0.001	<0.001	0.001
Cardiovascular disease										
n with influenza	187	36	287	381	463	187	36	287	381	463
n other diagnoses	20,661	16,264	229,386	286,185	392,253	20,661	16,264	229,386	286,185	392,253
% severe outcomes with influenza (95% CI)	3.7% (1.0–6.5)	5.6% (0.0–13.0)	4.2% (1.9–6.5)	4.7% (2.6–6.8)	1.5% (0.4–2.6)	13.9% (8.9–18.9)	13.9% (2.6–25.2)	18.5% (14.0–23.0)	17.8% (14.0–21.7)	24.8% (20.9–28.8)
% severe outcomes with other diagnoses (95% CI)	4.1% (3.8–4.4)	1.8% (1.6–2.0)	1.0% (1.0–1.1)	0.9% (0.9–1.0)	0.7% (0.7–0.7)	26.2% (25.6–26.8)	12.8% (12.3–13.3)	7.5% (6.6–6.8)	10.2% (10.1–10.3)	16.7% (16.6–16.8)
p-value	0.999	0.132	<0.001	<0.001	0.051	<0.001	0.802	0.508	<0.001	<0.001
COPD										
n with influenza	366	168	206	173	367	366	168	206	173	367
n other diagnoses	22,795	12,801	16,592	24,907	91,802	22,795	12,801	16,592	24,907	91,802
% severe outcomes with influenza (95% CI)	1.4% (0.2–2.6)	0.0% (0.0–2.2)	3.4% (0.9–5.9)	3.5% (0.7–6.2)	0.5% (0.0–1.3)	0.8% (0.0–1.7)	0.0% (0.0–2.2)	3.4% (0.9–5.9)	15.0% (9.7–20.3)	15.8% (12.1–19.5)
% severe outcomes with other diagnoses (95% CI)	0.3% (3.8–4.4)	0.4% (0.3–0.5)	1.3% (1.1–1.5)	0.8% (0.7–0.9)	0.5% (0.5–0.6)	0.4% (0.3–0.5)	0.6% (0.4–0.7)	4.8% (4.4–5.1)	10.3% (9.9–10.7)	16.2% (16.0–16.5)
p-value	0.004	0.999	0.018	0.003	0.714	0.196	0.999	0.508	0.045	0.887
Immunodeficiency										
n with influenza	26	3	1	0	0	26	3	1	0	0
n other diagnoses	1,044	1,046	385	101	72	1,044	1,046	385	101	72
% severe outcomes with influenza (95% CI)	0.0% (0.0–12.9)	0.0% (0.0–56.1)	100.0% (21.6–100.0)	N/A	N/A	7.7% (0.0–17.9)	0.0% (0.0–56.1)	100.0% (21.6–100.0)	N/A	N/A
% severe outcomes with other diagnoses (95% CI)	1.2% (0.6–1.9)	0.5% (0.1–0.9)	0.8% (0.0–1.7)	1.0% (0.0–2.9)	0.0% (0.0–5.7)	9.6% (7.8–11.4)	3.9% (2.7–5.1)	13.5% (10.1–16.9)	22.8% (4.6–31.0)	22.2% (12.6–31.8)
p-value	0.999	0.999	0.008	0.999	0.999	0.999	0.999	0.135	0.999	0.999

CI, confidence interval; COPD, chronic obstructive pulmonary disease; ICU, intensive care unit; N/A, not applicable. For each comorbidity, the denominator (n) of the percentages is the number of patients with influenza + the considered comorbidity or the number of patients without influenza + the considered comorbidity.

## Discussion

We used data from two large administrative health databases in Brazil and Mexico to estimate the number of hospitalizations with influenza, and to determine the role of age and comorbidities on the course of severity, based on ICD10 code discharge information. We found that the number of hospital admissions with influenza followed a seasonal profile, with specific periods presenting a peak of admissions for each country. The proportion of in-hospital deaths increased with increasing age in both countries (from 0.4% to 12.6% in Brazil and from 1.3% to 23.3% in Mexico). In Brazil, ICU admissions were higher in adults aged ≥50 years, and further increased in the presence of comorbidities, especially cardiovascular disease. In Mexico, comorbidities increased the risk of ICU admission and in-hospital CFR mostly in children.

The proportions of ICU admissions in our study (3.3% [95% CI 3.2–3.3] in Brazil and 1.8% [95% CI 1.6–2.0] in Mexico) were lower than those reported in the USA (14%–19%), whereas the total rates of in-hospital death (4.6% [95% CI 4.5–4.7] in Brazil and 6.9% [95% CI 6.5–7.2] in Mexico) were in the same range as those reported in the USA(<4.7%) and Spain (4.8%), but lower than in Costa Rica (12%) [18–20]. These variations may reflect the differences in healthcare seeking behavior of patients, hospital capacity and medical healthcare management in these countries. An estimation published by Borja *et al*. using the United States Center for Disease Control (US-CDC) method showed that the percentage of patients seeking healthcare services in the USA during the 2009–2010 pandemic influenza season was around 50% of patients covered by a major public healthcare provider [21]. In non-pandemic season the proportion seeking healthcare services may be even lower, with the Mexican Ministry of Health estimating as few as 1 in 10 patients seeking healthcare services [22]. Additionally, this unexpectedly low number of ICU admissions in those ≥65 years could be also due to an ICD-10 code reporting bias, as more severe influenza cases may evolve to pneumonia and be preferentially reported using that code, as suggested by the frequent use of the J12.9 code (unspecified viral pneumonia) in our data (31.1% in Brazil and 63.8% in Mexico). Global estimates on the number of hospitalizations with influenza have been recently reported in a review of influenza-associated lower respiratory tract infections and hospitalizations among adults and reported a substantial number in the region of the Americas, which includes Brazil and Mexico, as high as 137 (95% CI 80–217) influenza-associated lower respiratory tract infection (LRI) hospitalizations per 100,000 population [23]. Estimates are particularly high for young children, older adults, and those with underlying conditions.

In our study, in addition to providing local influenza admissions estimates, we have observed a role of age on the risk of severe outcomes in patients hospitalized with influenza, notably increased risks in those <5 years and those ≥50 years. This finding is consistent with a study in Chile showing that the risk of serious influenza illness was approximately 6 times higher in children <5 years of age and 13 times higher in adults ≥65 years of age compared to individuals between 5 and 64 years of age [24], or with observations from China, Hong Kong, Singapore, and Costa Rica, where most influenza-associated deaths occurred among older adults [20, 25]. While Brazil and Mexico are both upper-middle income countries, these results are also similar to data from administrative registries in high-income countries such as the USA, England, and Spain [18, 19, 26–28].

In addition to the role of age, the presence of comorbidities increased the duration of hospital stay, the risk of ICU admission, and death. Our results are aligned with recent studies demonstrating that comorbidities such as cardiovascular disease and COPD increase the risk of severe influenza outcomes. In England, 72% of influenza-attributable deaths in hospital occurred in adults ≥65 years of age with comorbidities. Also, the presence of comorbidities increased the admission rate by 1.8 fold in adults ≥65 years of age (from 0.46 to 0.84 per 1000) and by 5.7 fold in children 5–14 years of age (from 0.1 to 0.56 per 1000) [28].

Admission to ICU, in-hospital CFR estimates and any comparisons with other countries should be interpreted in light of the vaccine coverage rates (VCR) reported in these countries during the study period. According to data published by the Pan American Health Organization (PAHO), the influenza VCR among the elderly population in 2017 was 88% in Brazil and 94% in Mexico. In the pediatric population, the VCRs were 72% for Brazil and 84% for Mexico, respectively [29]. These differences of VCR may have affected the severity of the disease in some cases, impacting in some way the frequency of hospitalizations and ICU admissions in our study.

In Brazil, the epidemic period ranged from April to July whereas in Mexico peaks of hospital admissions with influenza by year ranged from January to the end of February. This observation is consistent with epidemiological surveillance data showing that influenza epidemics occur during winters in temperate regions (i.e Mexico), and often during the rainy season or all-year-round in tropical regions (i.e Brazil) [30–32]. The recent study from Caini et al. demonstrated the substantial heterogeneity of spatio-temporal patterns of influenza epidemics in Latin American countries, including Brazil [33].

The strengths of this study are that the analyses were conducted using two very large hospital databases over several consecutive influenza seasons. These databases have not been explored for the estimation of hospital admissions with influenza before, but has previously been done with dengue for example [10–12]. We also defined influenza cases using a conservative approach, based on ICD-10 codes specific for laboratory-confirmed influenza virus and viral pneumonia, and therefore the number of admissions are highly specific to these laboratory confirmations. However, the results and estimates of this study should be interpreted in light of several limitations. These two databases are primarily designed for administrative or reimbursement purposes, therefore the number of admissions with influenza may have been underestimated as patients are not always diagnosed with influenza, nor is it routinely laboratory-confirmed during hospitalization [26]. With no laboratory confirmation, admissions due to influenza may have been coded as pneumonia or other respiratory diseases. Also, the numbers of admissions with influenza were defined based on discharge codes which did not include respiratory or cardiovascular complications due to influenza. Influenza is not always prioritized in discharge codes when facing multiple comorbidities. The association between respiratory infections, especially influenza, and acute myocardial infarction for instance has been reported to be significant and responsible for hospitalizations [34, 35], and so this may have biased the true estimates of hospitalizations with influenza. Finally, from January to April 2010, the two databases may have included hospitalizations with the pandemic A(H1N1)pdm09 influenza virus because it became a seasonal virus only after this period. However, this period represents a small fraction of the results in the analyses and we did not see more hospitalizations during this period than during the following years. Direct comparison of severe outcome estimates between Brazil and Mexico is limited due to several differences between the countries. There are differences in ICD coding practices with the number of comorbidities reported for a given hospital admission in Brazil limited to one principal diagnosis and only one secondary diagnosis between 2010 and 2014, whereas multiple comorbidities could be reported for Mexico, which may explain the lower prevalence of comorbidities reported in Brazil compared with Mexico (1.0% vs 14.3%), especially in adults ≥65 years of age (1.7% in Brazil vs 50.7% in Mexico). Also, the database in Mexico captured 40% of admissions in the country and only from public hospitals, whereas in Brazil the database covered approximately 75% of admissions. Finally, differences including hospital management and health seeking behaviors preclude direct comparison between the countries. In Mexico, most admissions were recorded for young children, whereas, in Brazil, admissions were equally recorded in all age groups which can be due to differences for age prioritization in health care systems.

In conclusion, despite important limitations, the SIH/SUS and SAEH administrative hospital databases are useful to support estimations of number of hospitalizations with influenza and describe the associated severe outcomes. Developing linkage capacities between virological laboratories for the confirmation of influenza cases, private and public hospitals administrative databases could improve the estimates of hospitalizations associated to influenza at a national level. The cumulative role of age and comorbidities, especially cardiovascular disease, and their association with more severe outcomes in patients hospitalized with influenza is important, and should be further analyzed in prospective surveillance studies designed to support vaccination strategy decisions.

## Supporting information

S1 Table Duration of stay by age and predefined comorbidity in Brazil and Mexico. (TIF)

## References

[1] *World Health Organization*. Influenza (Seasonal). 2018 [10 Jan 2020]; Available from: https://www.who.int/news-room/fact-sheets/detail/influenza-(seasonal).

[2] Vaccines against influenza WHO position paper—November 2012. Wkly Epidemiol Rec, 2012. 87(47): p. 461–76. 23210147

[3] Coleman B.L., et al., Risk factors for serious outcomes associated with influenza illness in high- versus low- and middle-income countries: Systematic literature review and meta-analysis. Influenza Other Respir Viruses, 2018. 12(1): p. 22–29. 29197154 10.1111/irv.12504 PMC5818335

[4] Commission E., State of Health in the EU Companion Report 2019, in State of Health in the EU. 2019, European Commission: Luxembourg.

[5] Savy V., et al., Burden of influenza in Latin America and the Caribbean: a systematic review and meta-analysis. Influenza Other Respir Viruses, 2013. 7(6): p. 1017–32. 23210504 10.1111/irv.12036 PMC4634294

[6] Freitas F.T., et al., Influenza-associated excess mortality in southern Brazil, 1980–2008. Epidemiol Infect, 2013. 141(8): p. 1731–40. 23040669 10.1017/S0950268812002221 PMC9151596

[7] Salto-Quintana J.N., et al., Post-pandemic influenza-associated mortality in Mexico. Pathog Glob Health, 2019. 113(2): p. 67–74. 30895882 10.1080/20477724.2019.1589211 PMC6493299

[8] Taylor G., et al., Epidemiological features of influenza in Canadian adult intensive care unit patients. Epidemiol Infect, 2016. 144(4): p. 741–50. 26384310 10.1017/S0950268815002113 PMC4762243

[9] Lina B., et al., Complicated hospitalization due to influenza: results from the Global Hospital Influenza Network for the 2017–2018 season. BMC Infect Dis, 2020. 20(1): p. 465. 32615985 10.1186/s12879-020-05167-4 PMC7330273

[10] Macias A.E., et al., Real-World Evidence of Dengue Burden on Hospitals in Mexico: Insights from the Automated Subsystem of Hospital Discharges (Saeh) Database. Rev Invest Clin, 2019. 71(3): p. 168–177. 31184332 10.24875/RIC.18002681

[11] Werneck G.L., et al., Comorbidities increase in-hospital mortality in dengue patients in Brazil. Mem Inst Oswaldo Cruz, 2018. 113(8): p. e180082. 30043823 10.1590/0074-02760180082 PMC6056917

[12] Werneck G.L., et al., Mortality among hospitalized dengue patients with comorbidities in Mexico, Brazil, and Colombia. Am J Trop Med Hygiene, 2021 (in press). 10.4269/ajtmh.20-1163 PMC8274750 33970884

[13] Ministério da Saúde. Sistema de Informações Hospitalares do SUS. 2020 [09 Mar 2020]; Available from: http://sihd.datasus.gov.br/.

[14] ENSANUT. Encuesta Nacional de Salud y Nutrición, Resultados Nacionales. 2012; Available from: https://ensanut.insp.mx/informes/ENSANUT2012ResultadosNacionales.pdf.

[15] World Health Organization. International Statistical Classification of Diseases and Related Health Problems 10th Revision (ICD-10)-WHO Version for 2016. 2016 [12 Mar 2020]; Available from: https://icd.who.int/browse10/2016/en.

[16] World Health Organization. Manual and a Supplement for Estimating Disease Burden Associated With Seasonal Influenza. 2015; Available from: https://www.who.int/influenza/resources/publications/manual_burden_of_disease/en/.

[17] Serfling R.E., Methods for current statistical analysis of excess pneumonia-influenza deaths. Public Health Rep, 1963. 78(6): p. 494–506. 19316455 PMC1915276

[18] San-Roman-Montero J.M., et al., Inpatient hospital fatality related to coding (ICD-9-CM) of the influenza diagnosis in Spain (2009–2015). BMC Infect Dis, 2019. 19(1): p. 700. 31390988 10.1186/s12879-019-4308-5 PMC6686565

[19] Reed C., et al., Estimating influenza disease burden from population-based surveillance data in the United States. PLoS One, 2015. 10(3): p. e0118369. doi: 10.1371/journal.pone.0118369 25738736 PMC4349859

[20] Saborio G.G., et al., Influenza-associated Hospitalizations and Deaths, Costa Rica, 2009–2012. Emerg Infect Dis, 2014. 20(5): p. 878–81. 24750897 10.3201/eid2005.131775 PMC4012819

[21] Reed C., et al., Estimates of the prevalence of pandemic (H1N1) 2009, United States, April-July 2009. Emerg Infect Dis, 2009. 15(12): p. 2004–7. 19961687 10.3201/eid1512.091413 PMC3375879

[22] Borja Aburto V.H., et al., [Estimating the incidence of 2009 pandemic influenza A(H1N1) among IMSS affiliates]. Gac Med Mex, 2011. 147(4): p. 303–10. 21894228

[23] Lafond K.E., et al., Global burden of influenza-associated lower respiratory tract infections and hospitalizations among adults: A systematic review and meta-analysis. PLoS Med, 2021. 18(3): p. e1003550. 33647033 10.1371/journal.pmed.1003550 PMC7959367

[24] Sotomayor V., et al., Estimating the burden of influenza-associated hospitalizations and deaths in Chile during 2012–2014. Influenza Other Respir Viruses, 2018. 12(1): p. 138–145. 29446231 10.1111/irv.12502 PMC5818356

[25] Feng L., et al., Influenza-associated mortality in temperate and subtropical Chinese cities, 2003–2008. Bull World Health Organ, 2012. 90(4): p. 279–288B. 22511824 10.2471/BLT.11.096958 PMC3324869

[26] Ortiz J.R., et al., Influenza pneumonia surveillance among hospitalized adults may underestimate the burden of severe influenza disease. PLoS One, 2014. 9(11): p. e113903. 25423025 10.1371/journal.pone.0113903 PMC4244176

[27] Thompson W.W., et al., Influenza-associated hospitalizations in the United States. JAMA, 2004. 292(11): p. 1333–40. 15367555 10.1001/jama.292.11.1333

[28] Cromer D., et al., The burden of influenza in England by age and clinical risk group: a statistical analysis to inform vaccine policy. J Infect, 2014. 68(4): p. 363–71. 24291062 10.1016/j.jinf.2013.11.013

[29] Organization, P.A.H. Influenza Vaccine Coverage in countries and territories of the Americas, 2005–2018. 2019 2019 [cited 2019; Available from: http://ais.paho.org/imm/influenzacoveragemap.asp.

[30] Tamerius J.D., et al., Environmental predictors of seasonal influenza epidemics across temperate and tropical climates. PLoS Pathog, 2013. 9(3): p. e1003194. doi: 10.1371/journal.ppat.1003194 23505366 PMC3591336

[31] Viboud C., Alonso W.J., and Simonsen L., Influenza in tropical regions. PLoS Med, 2006. 3(4): p. e89. 16509764 10.1371/journal.pmed.0030089 PMC1391975

[32] Hirve S., et al., Influenza Seasonality in the Tropics and Subtropics—When to Vaccinate? PLoS One, 2016. 11(4): p. e0153003. 27119988 10.1371/journal.pone.0153003 PMC4847850

[33] Caini S., et al., Characteristics of seasonal influenza A and B in Latin America: Influenza surveillance data from ten countries. PLoS One, 2017. 12(3): p. e0174592. 10.1371/journal.pone.0174592 PMC5367818 28346498

[34] Kwong J.C., Schwartz K.L., and Campitelli M.A., Acute Myocardial Infarction after Laboratory-Confirmed Influenza Infection. N Engl J Med, 2018. 378(26): p. 2540–2541. 10.1056/NEJMc1805679 29949484

[35] Warren-Gash C., et al., Laboratory-confirmed respiratory infections as triggers for acute myocardial infarction and stroke: a self-controlled case series analysis of national linked datasets from Scotland. Eur Respir J, 2018. 51(3). 10.1183/13993003.01794-2017 PMC5898931 29563170

