# Supplementary figures and images for: Hospital admissions with influenza and impact of age and comorbidities on severe clinical outcomes in Brazil and Mexico
Source: PLoS One. 2022 Nov 10;17(11):e0273837. doi: 10.1371/journal.pone.0273837 (PMC9648751; doi:10.1371/journal.pone.0273837)

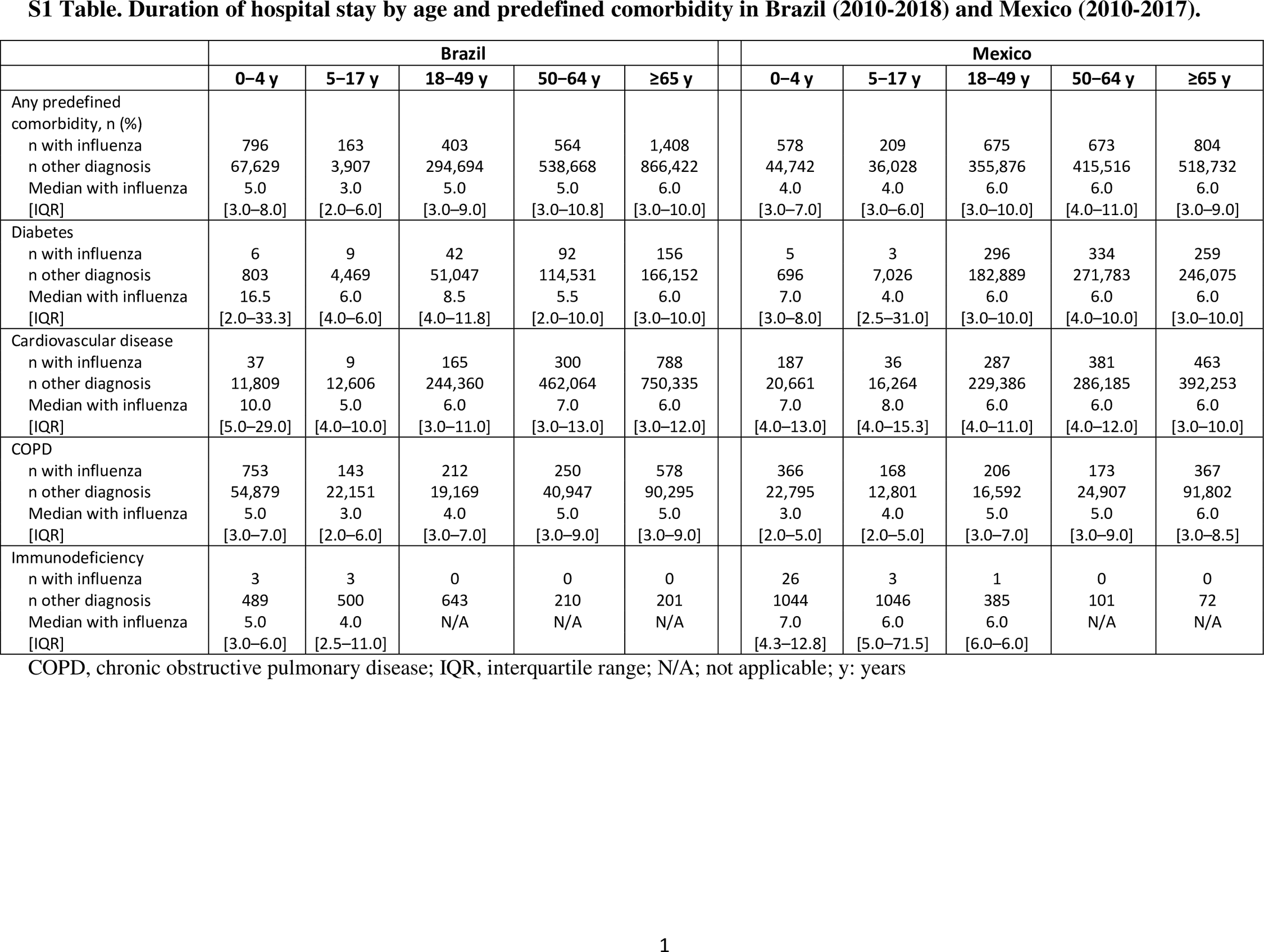

Supplement: S1 Table — (TIF) [file pone.0273837.s001.tif]
